# Behavioral and neuromorfological feaches indused by chronic stress in Q31Lmice, model of affective disorders

**DOI:** 10.1192/j.eurpsy.2026.11274

**Published:** 2026-07-31

**Authors:** T. Amstislavskaya, K. Smirnova, L. Smirnova

**Affiliations:** 1 Scientific Research Institute of Neurosciences and Medicine, Novosibirsk; 2 Laboratory of Molecular Genetics and Biochemistry, Research Institute of Mental Health, Tomsk National Research Medical Center, Tomsk, Russian Federation

## Abstract

**Introduction:**

Encoding a key ‘hub’ scaffolding protein, the ‘Disrupted-In-Schizophrenia-1’ (DISC1) gene has been strongly implicated in brain development and functions. Genetic variance in this gene is associated with major neuropsychiatric disorders, including schizophrenia, bipolar disorder, and major depression. DISC1 is abundantly expressed in the brain of humans and various model organisms. In mice, mutation Q31L in the *Disc1* gene reduces binding of DISC1 protein to GSK-3 (glycogen synthase kinase-3 alpha) and PDE4B (3’,5’-cyclic AMP phosphodiesterase 4B), accelerates degradation of BMAL1, which is involved in the regulation of glucocorticoid synthesis in the adrenal glands and the sensitivity of glucocorticoid receptor target genes.

**Objectives:**

The aim of this study is to distribution of BMAL1 protein in brain regions involved in behavior regulation in mice with Q31L mutation in *Disc1* gene and to investigate the behavioral response to different duration of stress effects.

**Methods:**

The male mice C57BL/6 mice and those with a mutation in the *Disc1* gene - Q31L were kept under standard conditions, with a 12:12 light regime, and their behavior was studied in the “open field”, “social preference” and “forced swimming” tests. Immunohistochemical analysis was performed on frozen brain sections using primary antibodies against BMAL1 and secondary ones - Alexa fluor 488.

**Results:**

Mice carrying point mutation Q31L in second exon *Disc1* exhibit depression-like phenotype: increased floating time and decreased social interaction. In CA1 and lateral habenula of Q31L mice was observed decreased BMAL1 protein expression. Both of this areas, especially lateral habenula involved in affective disorders.

**Conclusions:**

By the fact that BMAL1, regulate oligodendrocite proliferation and migration it can be proposed that DISC1-GSK3-BMAL1 pathway is potential mechanism regulating myelination and involved in affective disorders pathogenesis, so this protein can act as a molecular target for the treatment of affective disorders.

The study is supported the Russian Science Foundation No. 23-75-00023.

**Disclosure of Interest:**

None Declared

